# Intersectional socioeconomic disparities in continuous smoking through pregnancy among pre-pregnant smokers in Sweden between 2006 and 2016

**DOI:** 10.1186/s12884-024-06647-0

**Published:** 2024-07-06

**Authors:** Sten Axelsson Fisk, Jannike Cassel, Mikael Rostila, Can Liu, Sol Pia Juárez

**Affiliations:** 1 Department of Clinical Sciences Lund, Obstetrics and Gynaecology, Lund University, BMC C14. Lund, Lund, 22185 Sweden; 2 Department of Obstetrics and Gynaecology, Ystad Hospital, Ystad, Sweden; 3 Department of Public Health Sciences, Stockholm University, Stockholm, Sweden; 4 Centre for Health Equity Studies (CHESS), Stockholm University/Karolinska Institutet, Stockholm, Sweden; 5 Department of Neurobiology, Care Sciences and Society (NVS), Aging Research Center (ARC), Karolinska Institutet/Stockholm University, Stockholm, Sweden; 6 Clinical Epidemiology Division, Department of Medicine, Karolinska Institutet, Solna, Stockholm, Sweden

**Keywords:** Epidemiology, Maternity care, Prenatal care, Women’s health issues

## Abstract

**Background:**

While well-established associations exist between socioeconomic conditions and smoking during pregnancy (SDP), less is known about social disparities in the risk of continuous SDP. Intersectional analyses that consider multiple social factors simultaneously can offer valuable insight for planning smoking cessation interventions.

**Methods:**

We include all 146,222 pregnancies in Sweden between 2006 and 2016 where the mother smoked at three months before pregnancy. The outcome was continuous SDP defined as self-reported smoking in the third trimester. Exposures were age, education, migration status and civil status. We examined all exposures in a mutually adjusted unidimensional analysis and in an intersectional model including 36 possible combinations. We present ORs with 95% Confidence Intervals, and the Area Under the Curve (AUC) as a measure of discriminatory accuracy (DA).

**Results:**

In our study, education status was the factor most strongly associated to continuous SDP among women who smoked at three months before pregnancy. In the unidimensional analysis women with low and middle education had ORs for continuous SDP of 6.92 (95%CI 6.63–7.22) and 3.06 (95%CI 2.94–3.18) respectively compared to women with high education. In the intersectional analysis, odds of continuous SDP were 17.50 (95%CI 14.56–21.03) for married women born in Sweden aged *≥* 35 years with low education, compared to the reference group of married women born in Sweden aged 25–34 with high education. AUC-values were 0.658 and 0.660 for the unidimensional and intersectional models, respectively.

**Conclusion:**

The unidimensional and intersectional analyses showed that low education status increases odds of continuous SDP but that in isolation education status is insufficient to identify the women at highest odds of continuous SDP. Interventions targeted to social groups should be preceded by intersectional analyses but further research is needed before recommending intensified smoking cessation to specific social groups.

## Background

In Sweden, 8.8% of women smoke three months before pregnancy but only 1.9% smoke in pregnancy week 32 [1]. Supporting smoking cessation among pregnant women is important to improve health outcomes for mothers and foetuses [2–7] and to reduce social disparities in perinatal health [8].

There is a well-documented socioeconomic gradient in smoking prevalence during pregnancy. Pregnant women with lower education levels tend to have higher smoking rates compared to those with higher education. Additionally, immigrants from low- and middle-income countries often show smoking rates approaching that of native women with low education with increasing duration of residency [9]. Moreover, existing research indicate that social factors such as maternal education status and migration status are important predictors of successful smoking cessation during pregnancy [10] but it is not clear whether pregnant smokers with social risk factors for continuous smoking should be offered intensified smoking cessation support [11].

Most previous reports on social disparities in risk of smoking adopt a unidimensional approach that only considers one social dimension at the time [10, 12, 13]. Such approaches are implicitly based on the assumption that the effect of low education on risk of continuous smoking is homogenous among people with different migration status or civil status. According to intersectionality theory, social factors interact and this intersectional interaction need to be considered when mapping health disparities [14]. Different stances towards the use of social categories exist within the realm of intersectional research. The categorical intersectionality claim that even imperfect social categories must be used to monitor and address health disparities while anti-categorical intersectionality claims that the use of social factors in public health can perpetuate and strengthen social inequalities [15]. One way to determine when social factors could be considered for individual level interventions is to assess the Discriminatory Accuracy (DA) [16, 17], that is a quantification of how well a risk factor can discriminate individuals that will experience a health hazard from those that will not.

In this paper we investigate sociodemographic risk factors for continuous smoking through pregnancy. We adopt an intersectional perspective that simultaneously consider several social dimensions. By doing so, we aim to further the understanding of the intersecting social factors that influence risk of continuous smoking throughout pregnancy and add evidence on whether distinct smoking cessation support should be offered to subgroups of pregnant smokers with lower chances of quitting smoking.

## Methods

### Data sources and study population

This study is based on data from the Swedish Medical Birth Register (MBR), that contains information from antenatal care and delivery wards on maternal and child health for all women giving birth in Sweden. This includes birthweight, maternal BMI, smoking habits, as well as comorbidities. The data is automatically transferred and coverage of the Medical Birth Register is estimated to be 97–99% during the last 20 years [18]. Sociodemographic data on education, income and civil status is retrieved from Longitudinal Integrated Database for Health Insurance and Labor Market Studies [19] and country of birth is retrieved from the Swedish Total Population Register [20].

**Fig. 1:**
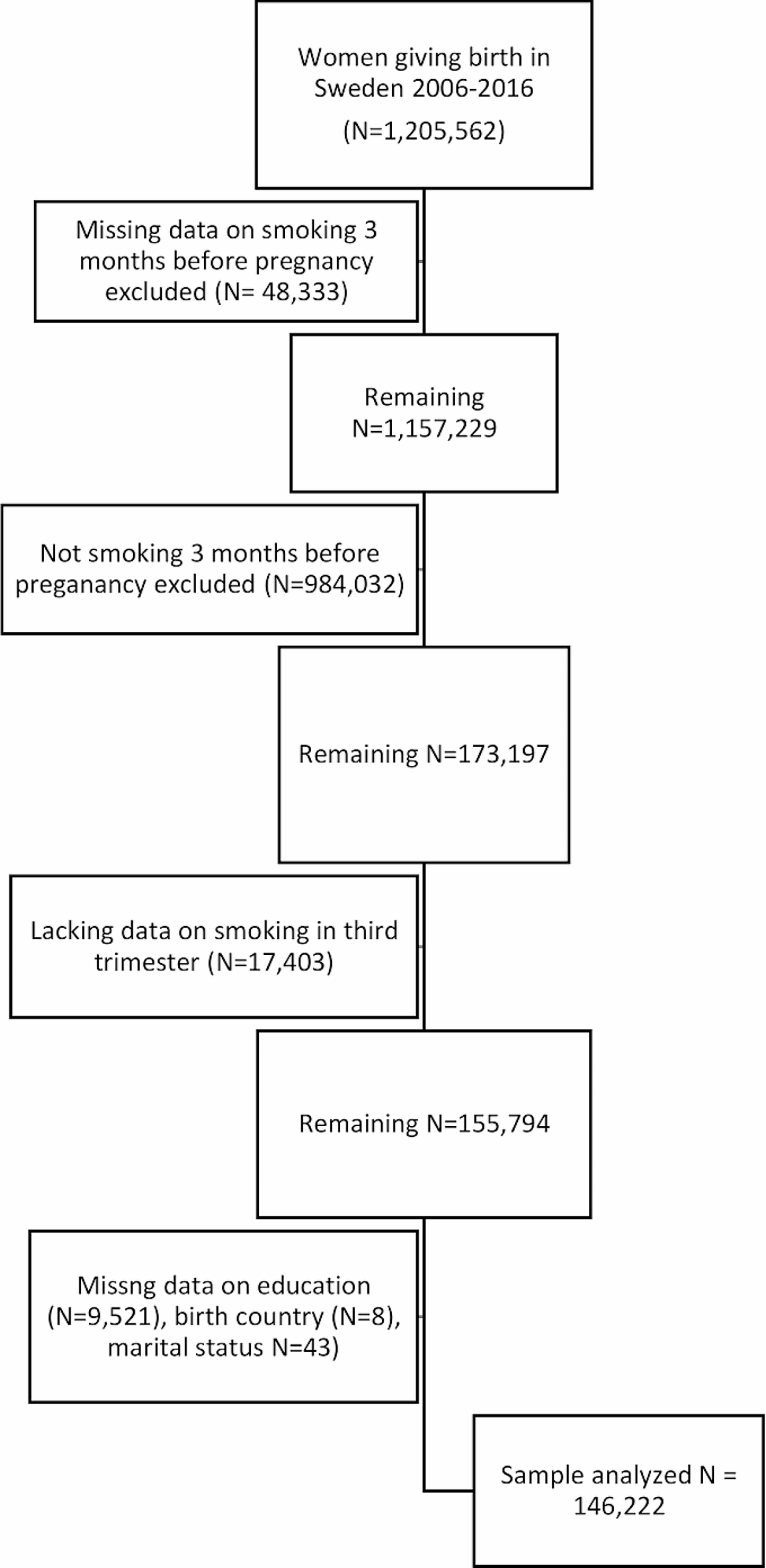
Flow chart of the study population

Starting with the 1,205,562 women giving birth in Sweden between 2006 and 2016, we excluded women with missing information on smoking status three months before pregnancy, women that did not smoke three months before pregnancy and women with lacking data on smoking status in the third trimester. We further excluded 9,572 women, 6.1%, with missing data on sociodemographic variables. 9,521 of these had missing information on education status. The study sample consists of 146,222 women that gave birth in Sweden between 2006 and 2016, 12.1% of the birthing population in this period, that smoked at three months before pregnancy and that had complete sociodemographic information and information on smoking status in the third trimester. The selection of the study population is presented in Fig. 1.

### Variables

The outcome variable is self-reported smoking status in the third trimester, this is documented by the midwife at the antenatal unit at inscription as well as in pregnancy week 30–32. The self-reported smoking status in the Medical Birth Register corresponds well with cotinine-levels in serum [21], shows expected associations to perinatal health outcomes [7] and has been used extensively in research. Our outcome variable is binary, those who reported smoking 1–9 cigarettes per day or more than 10 cigarettes per day were all defined as smokers, those who did not report smoking were defined as non-smokers.

Age was categorized into three categories; women aged 24 years or less, women 25–34 years old and women aged 35 years or older at the time of delivery. Education status was categorized into three categories: low (primary education, *≤* 9 years of education), middle (secondary education 10–14 years of education) and high (more than two years of postsecondary education, > 14 years of education). Migration status is categorized into people born in Sweden and people born outside Sweden (hereafter immigrants). Civil status is dichotomously categorized into people being married or living in registered partnership in one category and unmarried, divorced and widowed people in another category. These variables were then used to create the intersectional matrix.

### Statistical analysis

In a first step, we present the distribution of continuous smoking in pregnancy week 30–32 in the different sociodemographic groups (see Table 1). We then perform multiple logistic regression analyses in two consecutive models. Model 1 is mutually adjusted for all sociodemographic variables assessed in this study. In model 2, we created an intersectional matrix that consists of all unique combinations of these variables, resulting in 36 (3 × 3 × 2 × 2) intersectional strata that is the only exposure variable in this model. Since each unique combination exists in the matrix any potential intersectional interaction between the included variables will lead to improved performance in model 2 compared to model 1.

**Table 1:** Descriptive statistics and proportion of pregnant people still smoking by pregnancy week 32 among 146,122 women that smoked 3 months prior to pregnancy and gave birth in Sweden between 2006 and 2016

	Smoking week 30–32	
	**No**	**Yes**
	n (%)	n (%)
**Age**		
<=25	28,246 (66,89)	13,983 (33,11)
25–34	59,765 (71,08)	24,311 (28,92)
>=35	12,432 (62,42)	7,485 (37,58)
**Education**		
< 9 years	21,984 (53.41)	19,175 (46.59)
9–12 years	55,356 (70.58)	23,077 (29.42)
> 12 years	23,103 (86.76)	3,527 (13.24)
**Migration status**		
Immigrant	17,491 (67.90)	8,268 (32.10)
Swedish	82,876 (68.85)	37,494 (31.15)
**Civil status**		
Married	28,371 (68.98)	12,761 (31.02)
Not married	72,072 (68.58)	33,018 (31.42)

For all models, we calculate the area under the receiver operating characteristics curve (AUC) as a measurement of DA. The AUC value takes both sensitivity and specificity into account and is a measure of how accurately a model can separate pregnant women that will continue smoking during pregnancy from those that will not. The AUC can take a value between 0.5, which implies that the model does not provide more information than flipping a coin, and 1.0, which means that the model perfectly identifies women with continuous SDP. The following cut-off values were adopted as proposed in a previous study: (1) ‘absent or very small’ (AUC = 0.5–0.6) (2), ‘moderate’ (AUC > 0.6–≤0.7) (3), ‘large’ (AUC > 0.7–≤0.8) and (4) ‘very large’ (AUC > 0.8) [22].

Model 2, where all combinations of sociodemographic variables are represented and eventual interactions are captured, would perform better than model 1 if interactions would be present. An increase in AUC in model 2 thus indicates that statistical interaction between the included variables is occurring, whereas overlapping confidence intervals for the AUC-values of these models implies that such interaction is absent.t.

All analyses are performed using STATA® version MP 15.1 (StataCorp, College Station, TX).

## Results

The sociodemographic distribution of women that continued to smoke or stopped smoking during pregnancy is shown in Table 1. Of the women smoking at three months before pregnancy, continuous smoking was more common in the oldest (38%) and youngest (33%) age categories, compared to women aged 25–34 years (29%). There was an educational gradient in continuous smoking, 47% of women with < 9 years of education, 29% of women with 9–12 years of education and 13% of women with more than 12 years of education continued to smoke in the third trimester. 32% of immigrants and 31% of women born in Sweden continued to smoke. The percentage of continuous smokers were 31% both for married women and women that were not married.

Odds Ratios (ORs) and AUC-values from model 1 are shown in Table 2. In model 1, mutually adjusted for all sociodemographic variables as unidimensional factors, women aged < 25 had lower OR than women aged 25–34, and women aged 35 years or more had the highest OR. ORs were highest for women with < 9 years of education, women with 9–11 years of education had higher ORs than women with *≥* 12 years of education. Non-married had lower ORs of continuous smoking compared to married individuals but no difference was found in odds of continuous SDP between people born in Sweden and immigrants.

**Table 2:** Odds ratios from model 1 and AUC-values from model 1 and model 2

Variable	Model 1	Model 2
	OR (95%CI)	Intersectional
**Age**		Odds Ratios from Model 5 in Table 3
<=25	0.82 (0.80–0.85)	
25–35	Ref	
>=36	1.70 (1.64–1.76)	
**Education**		
< 9 years	6.92 (6.63–7.22)	
9–12 years	3.06 (2.94–3.18)	
> 12 years	Ref	
**Migration status**		
Swedish	Ref	
Immigrant	1.03 (0.99–1.06)	
**Civil status**		
Married	Ref	
Not married	0.95 (0.93–0.98)	
**AUC (95%CI)**	0.658 (0.655–0.661)	0.660 (0.657–0.662)

Odds ratios (OR) and 95% Confidence intervals (CI) from model 1 and AUC-values with 95% Confidence Intervals from model 1 and 2, results from logistic regression with unidimensional social risk factors as explanatory variables and continuous smoking at pregnancy week 32 as the outcome. Analysis performed among 146,222 women that smoked 3 months prior to pregnancy and gave birth in Sweden between 2006 and 2016

ORs from model 2, where the reference category was married women aged 25–34 that were born in Sweden and had high education, are presented in Table 3. The three strata with highest OR of continuous smoking included (1) women aged > 35 years with less than 9 years of education, born in Sweden that were married (OR 17.50, 95%CI 14.56–21.03), (2) women aged > 35 years with less than 9 years of education, born in Sweden that were not married (OR 16.30, 95%CI 14.17–18.75) and (3) immigrant women aged > 35 years with less than 9 years of education that were not married (OR 10.22, 95%CI 8.42–12.47). The three strata with lowest OR for continuous smoking in the third trimester were (1) 25–34 years old women that were born in Sweden, that were not married and had > 12 years of education, (OR 0.98, 95%CI 0.87–1.10) (2) 25–34 years old women, born in Sweden that were married and had > 12 years of education (reference group in regression analysis) and (3) women younger than 25 years that were born in Sweden, had more than 12 years of education and that were married (OR 1.15, 95%CI 0.73–1.81).

**Table 3:** Odds Ratios, number of individuals and numbers of still-smokers from intersectional model 2. Odds ratios (OR) and 95% Confidence Intervals (CI) from intersectional logistic regression analyses with all combinations of age, education, country of birth and civil status as the explanatory variables and continuous smoking during at pregnancy week 32 as the outcome. Analysis performed among 146,222 women that smoked 3 months prior to pregnancy and gave birth in Sweden between 2006 and 2016

Age			Education			Country of birth		Marital		OR (95%CI)			
*≤* 25	25–34	*≥* 35	Low	Middle	High	Sweden	Immigrant	Unmarried	Married		**Persons per stratum**	**Number of still-smokers (%)**	
		✔	✔			✔			✔	17.50 (14.56–21.03)	686	449 (65,45)	
		✔	✔			✔		✔		16.30 (14.17–18.75)	1587	1013 (63,83)	
		✔	✔				✔	✔		10.25 (8.42–12.47)	521	274 (52,59)	
	✔		✔			✔			✔	9.46 (8.41–10.63)	3295	1667 (50,59)	
	✔		✔			✔		✔		9.17 (8.27–10.16)	10,053	5008(49,82)	
		✔	✔				✔		✔	8.44 (7.05–10.10)	662	316 (47,73)	
✔			✔			✔			✔	8.20 (7.19–9.35)	1863	876 (47,02)	
	✔		✔				✔	✔		7.85 (6.87–8.97)	1791	823(45,95)	
	✔		✔				✔		✔	7.12 (6.28–8.08)	2313	1007 (43,54)	
✔			✔			✔		✔		6.74 (6.10–7.45)	16,031	6764 (19, 42)	
✔			✔				✔		✔	6.59 (5.62–7.72)	985	410 (41,62)	
		✔		✔		✔			✔	6.57 (5.81–7.43)	2596	1079 (41,56)	
		✔		✔		✔		✔		6.55 (5.88–7.31)	5668	2352 (41,50)	
✔			✔				✔	✔		6.53 (5.65–7.53)	1372	598 (4, 41)	
		✔		✔			✔	✔		6.15 (5.25–7.21)	988	395 (39,98)	
		✔		✔			✔		✔	5.58 (4.78–6.50)	1129	425 (37,64)	
	✔			✔			✔	✔		4.31 (3.82–4.85)	3337	1061 (31,80)	
	✔			✔			✔		✔	4.01 (3.57–4.50)	4225	1277 (22, 30)	
	✔			✔		✔			✔	3.93 (3.53–4.36)	9657	2880 (29,82)	
✔				✔		✔			✔	3.65 (3.19–4.17)	2194	621 (28, 30)	
	✔			✔		✔		✔		3.58 (3.24–3.95)	30,084	8396 (27,91)	
✔				✔			✔		✔	3.50 (2.95–4.14)	983	270 (27,47)	
✔				✔			✔	✔		3.45 (2.92–4.09)	996	271 (21, 27)	
✔				✔		✔		✔		2.99 (2.70–3.31)	16,576	4050 (24, 43)	
		✔			✔		✔		✔	2.88 (2.41–3.43)	960	228 (23,75)	
		✔			✔		✔	✔		2.25 (1.82–2.79)	683	134 (19,62)	
		✔			✔	✔		✔		2.20 (1.93–2.51)	2891	556 (19, 23)	
✔					✔		✔		✔	2.13 (1.48–3.07)	203	38 (18,72)	
	✔				✔		✔	✔		2.00 (1.71–2.33)	1743	310 (17,79)	
		✔			✔	✔			✔	1.90 (1.62–2.24)	1546	264 (17,08)	
	✔				✔		✔		✔	1.80 (1.57–2.07)	2835	463 (16, 33)	
✔					✔		✔	✔		1.25 (0.72–2.16)	126	15 (11,90)	
✔					✔	✔		✔		1.16 (0.90–1.49)	701	78 (11, 13)	
✔					✔	✔			✔	1.15 (0.73–1.81)	199	22 (11,06)	
	✔				✔	✔			✔	REF	4801	469 (9,77)	
	✔				✔	✔		✔		0.98 (0.87–1.10)	9942	950 (9,56)	

The AUC increases from 0.658 in model 1 by 0.002 units to 0.660 in the intersectional model 2, but the 95% confidence intervals were overlapping.

## Discussion

### Main findings

In this study, we report clear educational disparities in risk of continuous smoking through pregnancy in Sweden. The intersectional stratum including married women aged > 35 years with less than 9 years of education, born in Sweden had the highest odds of continuing smoking during pregnancy. In the unidimensional analysis, educational status has the strongest influence on odds of continuous smoking but age category also affected these odds. Civil status and migration status, as defined in our study, were less relevant for determining an individuals’ odds of continuing smoking during pregnancy. The overlapping confidence intervals of the AUC values between the two models indicate an absence of statistical interaction between the included variables.

The approach adopted in this study enables us to see both the variation between and within broad social categories. For example, while women aged < 25 had the lowest odds of continuous smoking, the intersectional analysis revealed that pregnant women in this age group that had low education, were married, and born in Sweden had an OR > 8 compared to the reference category despite their protective age category. All women with low education belonged to the intersectional strata with highest odds of continuous smoking. Within this group of women with low education, OR of continuous smoking compared to the reference category ranged from 6.53 (5.65–7.53) among young, immigrant women that were not married to 17.50 (14.56–21.03) among older, married, women born in Sweden. This illustrates the utility of an intersectional perspective even when mapping an outcome like continuous smoking through pregnancy that is strongly associated to one social dimension, namely education status.

The intersectionality theory has its roots in gender studies and have traditionally adopted qualitative research methods [23]. The translation to quantitative population health research comes with controversies, for example regarding the use of crude social categories [15] and difficulties in measuring norms and experiences of oppression [23] in register studies. Intersectional scholars may have different views on whether this study should qualify as intersectional or not. We claim that this sort of research inspired by intersectionality theory can enrich population health by deepening understanding of how health is affected among people experiencing multiple social disadvantages. Furthermore, to challenge the relevance of broad categories such as “immigrants”, “single mothers”, “young mothers” or stratifications of education status is another reason to perform quantitative research inspired by intersectionality theory. The assessment of the DA is one part of the critical stance towards socially defined categories but is also crucial for determining the clinical relevance of sociodemographic risk factors. In our study, the ORs were high for some groups but the DA was moderate. This cautions against proposals of exclusive targeting of health care interventions towards groups with increased average risk of continuous smoking. Intersectionality theory is a critical theory aiming at eliminating structural causes of inequities in health [23]. Therefore, rather than targeted interventions to individuals with social risk factors, political actions to ensure equitable education should be the first priority.

### Relation to previous studies

The educational gradient that we found was similar to a Norwegian study that investigated chances of quitting smoking in the third trimester among pregnant women that smoked in the beginning of pregnancy [24]. A European study based on an online-survey found that low educational level and living alone were determinants of continuous smoking during pregnancy among women smoking before pregnancy [25]. In a systematic review from 2010, low education and being non-white / immigrant were factors negatively correlated with successful smoking cessation in the included studies [26]. Previous results are conflicting with regards to whether high age is protective or a risk factor for continuous SDP, according to a meta-analysis [10]. Teenage pregnancy is associated to both low socioeconomic position [27] and early smoking initiation [28] which are in turn associated to higher risk of continuous SDP. In our study, younger mothers had the lowest odds of continuous SDP, but we did not isolate teenagers in any specific age category. Nicotine dependence increases with duration of smoking, and is thus associated with more advanced maternal age. This is a possible explanation of our finding of increased risk of continuous SDP in the older age categories.

In a previous intersectional study of smoking prevalence in the general Swedish population, i.e. both pregnant and non-pregnant, using the same methodology, a similar pattern of smoking prevalence was found albeit civil status and migration status had a clear effect on smoking status in that study, whereas in our study specifically focusing on women who smoked at three months before pregnancy those variables were not strongly associated to increased odds of continuous smoking [22].

We have not identified any previous studies adopting an intersectional approach to study risk of continuous smoking in late pregnancy among women smoking before conception. The intersectional mapping of the sociodemographic distribution of continuous smoking is therefore novel, as well as the finding of no intersectional interaction. As far as we know, no previous studies exist that report the DA of social or other risk factors to identify pre-pregnancy smokers at increased risk of continuous smoking. To put the AUC-value of 0.66 in perspective, a previous study examining the determinants of Small for Gestational Age in a similar population and including the same risk factors reported an AUC-value of 0.57 [29]. Early pregnancy BMI had an AUC-value of 0.55 to predict caesarean section, 0.71 to predict macrosomia and 0.66 to identify dystocia in a Polish population [30]. A model including pre-pregnancy BMI *≥* 30 had an AUC-value of 0.66 to predict infants born Large for Gestational Age in a Swedish study. Clinical risk factors and universal HbA1c had AUC-values of 0.72 and 0.75 respectively to predict gestational diabetes mellitus [31].

### Strengths and limitations

This is a study based on a large database including 146,222 pregnancies that represent a very large proportion of all pregnancies between 2006 and 2016 in Sweden. The sociodemographic data is of high quality and has been validated for medical research [20].

A limitation of this study is that 10% were excluded due to lacking information on smoking status in the third trimester. In the Medical Birth Register, the proportion of missing information on smoking in the third trimester has declined from 27% in 2006 to 12% in 2016, with large disparities between different counties. We do not know the reasons for missing information, but since the problem has prevailed regardless of whether electronic or manual transferring of the information it is unlikely to be technical [32]. Either the midwives have not asked the pregnant woman or abstained from registering the information. If the risk of not registering is higher in the case of status quo – the woman continues smoking, or in the scenario when the woman has ceased smoking and thus do not require further interventions is unclear, therefore we cannot assess the direction of the bias introduced. In addition, the fact that the smoking is based on self-reported information rather than any objective measurement implies a risk of bias due to measurement error, both for those excluded due to erroneously declaring that they were non-smokers before pregnancy and for the outcome. A validation study indicated that 95% of non-smokers in the MBR were true non-smokers based on serum-cotinine levels and that 88% of self-reported smokers in first trimester were also smokers at the time of delivery. These figures suggest that the risk of significant measurement error in this study is low [21]. There is conflicting evidence in relation to whether validations are contingent on socioeconomic position. While a study from the USA reported that self-reported smoking status was less accurate among people with higher education than among people with lower education [33], no such pattern was found in a Finnish context [34]. In order to not end up with too few cases in the intersectional strata, we used a dichotomous definition of smoking both at three months before pregnancy and during the third trimester despite evidence that the intensity of smoking influences perinatal outcomes such as birth weight [7]. We chose not to include Swedish smokeless tobacco (snus) in this paper since it is not as relevant for perinatal outcomes such as birth weight [35]. We unfortunately have a relatively high proportion of missing education, which is more common among migrants. If this information is not missing at random, our complete-case analysis could be biased. If the excluded individuals had low education status, which is more common among immigrants, and higher rates of continuous SDP, it is possible that we underestimated the true association between low education status and continuous SDP.

A problem confronted when performing research inspired by intersectionality theory that aims at providing guidance for public health and clinical interventions is the complexity arising when simultaneously considering several social dimensions. While from a statistical point of view, a large number of intersectional strata can be handled [36], this does not overcome the clinical problem that requires sufficiently simple categorizations in order to be useful. Therefore, one must balance the need for a sufficiently small number of intersectional strata with the poor precision that comes with crude categorizations. For example, our binary categorization of civil status can be criticized for grouping separated, widowed but also un-married people that live together into the same category as “not married” people. We claim that presently being married or in a registered relationship is a proxy for a stable relationship and thus grasps some aspects of normativity. Socioeconomic position can be operationalized using proxy variables such as education status, individual income, neighbourhood deprivation indices, occupational status or composite measurements [37]. None of the proxy variables are exhaustive in the sense that they capture all aspects of socioeconomic position. Education status captures broad aspects of socioeconomic position since it exerts its effect on health through material, psychosocial and behavioural pathways. The main reason for choosing education status is that we consider it the socioeconomic factor that is most feasible to ask about in a clinical setting. In addition to this, education status is less affected by temporal fluctuations during reproductive years compared to other indices of socioeconomic position. By measuring education solely in terms of time spent in education, we may underestimate the long-term educational status of individuals who pursue higher education later in life [38]. This issue is particularly important among younger pregnant women.

The dichotomous definition of migration status into immigrants and people born in Sweden leads to a large heterogeneity within the group of immigrants. Immigrants maintain smoking patterns of their previous countries [39] and smoking rates increase with the length of stay in Sweden until reaching the smoking prevalence of people born in Sweden with low education and low income, with the exception of women from countries with very high human development index that showed higher smoking rates than women born in Sweden [9].

### Implications and future research

Recent perinatal guidelines in Sweden recommend interventions such as thromboprophylaxis [40] or induction of labour [41] for patients with “low socioeconomic status” or “origin from Subsaharan Africa”. We argue that an intersectional stance is critical when considering social targeting in perinatal care. Our results have two principal implications for efforts to reduce socioeconomic inequalities in the risk of continuous SDP. According to both the unidimensional and intersectional analyses, low education is the social risk factor most strongly associated to risk of continuous SDP, compared to migration background, age or marital status. This means that low education is associated with continued SDP across all origins, age categories and marital status. However, the intersectional analysis shows that the odds of continuing SDP are higher when low education is combined with other social categories. This combination is outcome specific in the sense that the intersection with the highest risk of continued smoking is not necessarily the intersection between the most vulnerable categories across the variables considered. For example, in our study, older Swedish-born women with low education are more likely to continue smoking than older foreign-born women with low education. Taken together, this indicates that smoking cessation efforts among women with low education could have positive effects in different subgroups, although some subgroups may benefit more than others. This finding, combined with a moderate AUC value of 0.66, suggests that smoking cessation interventions for women with low education should be considered with caution, as the risk of stigmatisation may be more detrimental than the benefits of smoking cessation. This is particularly important as psychosocial stress is particularly harmful for pregnant women. In this study we focused on the total effect of the social factors on odds of continuous smoking in the third trimester among women that smoked at three months before pregnancy. There are certainly unmeasured differences between the women with low education or older age, that displayed increased odds of continuous SDP, that explain these risk differences. Future studies that also target mediators of these associations could provide valuable information to guide antenatal smoking cessation interventions, such factors include discrimination, parity [26], pregnancy intention [42], health literacy [43] and depression [44].

According to a systematic review, the effectiveness of smoking cessation support can be enhanced with more intensive smoking cessation counselling outside standard antenatal care setting or when offering feedback or financial incentives [8]. Non-pregnant daily smokers in Sweden are recommended *qualified smoking cessation counselling*, i.e. referral to a smoking cessation specialist. The National Board of Health suggests that pregnant smokers should be offered *smoking cessation counselling*, a less intensive intervention than qualified counselling, due to a lack of evidence of improved smoking cessation results with qualified counselling compared to standard counselling and the higher cost of qualified smoking cessation counselling. Offering qualified smoking cessation, with authorized interprets when indicated, to women with increased risk of continuous smoking based on the social risk factors presented in this study is a possible clinical intervention that could be explored in future studies.

## Conclusions

There are clear socioeconomic differences in the odds of continuous smoking in the third trimester among women in Sweden that smoked three months before pregnancy, education status being the most important of factors included in this study according to both the unidimensional and intersectional models. If intensified smoking cessation should be offered to a socially defined group, it should be directed to pregnant women with a low education. The heterogeneity in odds of continuous SDP even in this group, together with the moderate Discriminatory Accuracy, implies that potential harming side effects of socially targeted interventions must be ruled out before recommending such targeted interventions.

## Data Availability

The data that this study is based on are available from the Swedish National Board of Health and Welfare, and Statistics Sweden. However, register data are protected by rules of confidentiality. Researchers can only access the data after a special review that includes ethical approval from the authorities that control the data and from regional Ethics Committees. Swedish authorities do not provide individual-level data to researchers based in other countries. Researchers in other countries are instead advised to collaborate with Swedish colleagues in order to gain access to data.

## Abbreviations

AUC: Area Under the receiver operating characteristic Curve
CI: Confidence Interval
DA: Discriminatory Accuracy
MBR: The Swedish Medical Birth Register
OR: Odds Ratio
SDP: Smoking During Pregnancy
